# The Facts as to the Stamping out of Contagious Animal Diseases

**Published:** 1885-01

**Authors:** 


### THE FACTS AS TO THE STAMPING OUT OF CONTAGIOUS ANIMAL DISEASE.

The following is taken from a late number of the *Jersey Bulletin :*

> Prof. Salmon, the head of the Bureau of Animal Industry, who is preparing his first report, says that the bureau will not be a success until Congress gets rid of the ridiculous and exploded State rights theory, as far as it pertains to this subject. We are much hampered by the law, and there is no satisfaction in spending months of labor to find the disease attacking the stock-producing industry, and not to be able to get rid of it after we find it and definitely locate it. Pleuro-pneumonia is rapidly spreading in the West. We are unable to do more than coöperate with the State authorities. Without the aid of Congressional action, there is no hope.

The editor expresses surprise that Dr. Salmon should express the views he does, and that he will thereby "do more harm than all the pleuro-pneumonia."

The truth of the matter is, that Dr. Salmon is the first man in the position which he occupies, that has been man enough to tell the truth about the impossibility of stamping out contagious diseases in our animals if they obtain any great degree of dispersion in different herds in various States.

Over three years ago we stated that it would be impossible to do it in the United States, and we have been constantly stating it ever since, so long as the doctrine or principle of State rights had any recognition whatsoever.

We dogmatically assert that in spite of the utmost and most honest efforts of the United States government, no matter what money they spend, no matter if they kill every diseased animal in the country, and every one that is at present suspected to be diseased with the lung plague, that the Washington authorities can never stamp it out until the doctrine of State rights is absolutely abolished with regard to these questions, and we have one National Veterinary Police system for the country.

Britain has tried the same game. She would not listen to the advice of her best Veterinarians, but, like our government in the past, paid far more attention to what quacks and empirics had to say. The "rights of the British freeman could not be interfered with," when the fact is, the individual or individual state has no rights when, by their action or non-action, the whole home population of a country is endangered. Even to-day the British Government cannot get rid of the lung plague because of the non-support and "rights" of the local authorities. B.

